# An Insight into Advanced Approaches for Photosensitizer Optimization in Endodontics—A Critical Review

**DOI:** 10.3390/jfb10040044

**Published:** 2019-09-30

**Authors:** Patrícia Diogo, M. Amparo F. Faustino, M. Graça P. M. S. Neves, Paulo J. Palma, Isabel P. Baptista, Teresa Gonçalves, João Miguel Santos

**Affiliations:** 1 Institute of Endodontics, Faculty of Medicine, University of Coimbra, 3000-075 Coimbra, Portugal; 2 FMUC, Faculty of Medicine, University of Coimbra, 3000-370 Coimbra, Portugal; 3 QOPNA & LAQV-REQUIMTE and Chemistry Department, University of Aveiro, 3810-193 Aveiro, Portugal; faustino@ua.pt (M.A.F.F.); gneves@ua.pt (M.G.P.M.S.N.); 4 Institute of Periodontology, Faculty of Medicine, University of Coimbra, 3000-075 Coimbra, Portugal; 5 CNC, Center for Neuroscience and Cell Biology, University of Coimbra, 3004-504 Coimbra, Portugal

**Keywords:** antimicrobial photodynamic therapy, root canal disinfection, biofilms, endodontic therapy

## Abstract

Apical periodontitis is a biofilm-mediated disease; therefore, an antimicrobial approach is essential to cure or prevent its development. In the quest for efficient strategies to achieve this objective, antimicrobial photodynamic therapy (aPDT) has emerged as an alternative to classical endodontic irrigation solutions and antibiotics. The aim of the present critical review is to summarize the available evidence on photosensitizers (PSs) which has been confirmed in numerous studies from diverse areas combined with several antimicrobial strategies, as well as emerging options in order to optimize their properties and effects that might be translational and useful in the near future in basic endodontic research. Published data notably support the need for continuing the search for an ideal endodontic photosensitizer, that is, one which acts as an excellent antimicrobial agent without causing toxicity to the human host cells or presenting the risk of tooth discoloration. The current literature on experimental studies mainly relies on assessment of mixed disinfection protocols, combining approaches which are already available with aPDT as an adjunct therapy. In this review, several approaches concerning aPDT efficiency are appraised, such as the use of bacteriophages, biopolymers, drug and light delivery systems, efflux pump inhibitors, negative pressure systems, and peptides. The authors also analyzed their combination with other approaches for aPDT improvement, such as sonodynamic therapy. All of the aforementioned techniques have already been tested, and we highlight the biological challenges of each formulation, predicting that the collected information may encourage the development of other effective photoactive materials, in addition to being useful in endodontic basic research. Moreover, special attention is dedicated to studies on detailed conditions, aPDT features with a focus on PS enhancer strategies, and the respective final antimicrobial outcomes. From all the mentioned approaches, the two which are most widely discussed and which show the most promising outcomes for endodontic purposes are drug delivery systems (with strong development in nanoparticles) and PS solubilizers.

## 1. Introduction

Antimicrobial resistance arises when microorganisms which cause infection survive a drug exposure that would normally eradicate them or prevent their growth. Some strains develop resistance tools in order to survive in toxic environments, leading to the emergence of so-called superbugs and ESKAPE pathogens (*Enterococcus faecium*, *Staphylococcus aureus*, *Klebsiella pneumoniae*, *Acinetobacter baumannii*, *Pseudomonas aeruginosa* and *Enterobacter* species) [1].

It is recognized that bacterial microbiota in a healthy oral cavity are dissimilar from those in a diseased one. Furthermore, in oral disease, systemic infections have been related to oral sepsis since 1922; the oral cavity contains almost 1000 different species, with a predominance of streptococci [2]. Recently, oral bacteria and dental infections have been associated with the development of acute coronary thrombosis, which is the first major cause of worldwide death [3].

Modern health over the past century has been built upon the knowledge that diseases might be prevented or treated using antimicrobials; however, today it is recognized that chronic infections, such as persistent endodontic infections, are caused by a few persistent microorganisms. *Enterococcus faecalis* is strongly associated with endodontic failure, as are *Pseudomonas*, staphylococci, and streptococci [4,5]. Moreover, persistent endodontic infections are difficult to control because they are caused by microorganisms which are present in the primary infection and which managed to survive intracanal antimicrobial procedures and remain inside the root canal system or invade the periapical tissues [6,7]. The field of endodontics would thus benefit from better and more predictable root canal disinfection protocols. Considerable research effort has been invested into this topic, as shown by the numerous papers on root canal disinfection which have recently been published [8]. Since chronic and persistent endodontic infections are associated with biofilm growth, special attention is being given to studies involving experimental biofilms as substrates instead of classical planktonic suspensions [9]. 

**Figure 1:**
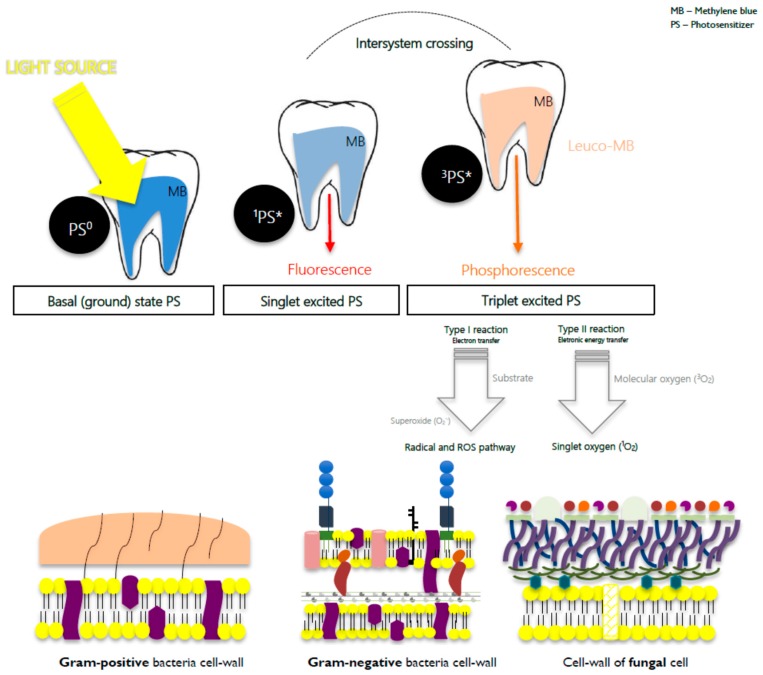
Antimicrobial photodynamic therapy (aPDT) photochemical mechanisms and its reactions products versus microorganisms’ cell wall. Teeth and microorganism cell-wall illustration sizes and scales are not respected. PS: photosensitizer; ROS: reactive oxygen species; *: excited state.

Antimicrobial photodynamic therapy (aPDT) has emerged as a current experimental approach for a broad spectrum of biofilm-mediated diseases, and is of particular interest in endodontics due to its simple approach, where a visible light source of a suitable wavelength, typically a light-emitting diode (LED), triggers a non-toxic agent, usually an innocuous dye when non-radiation is applied (i.e., photosensitizer (PS)), which is selectively absorbed by a target tissue. However, when activated and in the presence of molecular oxygen (^3^O_2_), it produces reactive oxygen species (ROS) like singlet oxygen (^1^O_2_), leading to the eradication of microorganisms without inducing resistance or damage to the host [10]; see Figure 1.

These species are able to interact with the cell wall, causing a reduction in membrane thickness, and thereby rendering the cells unable to withstand turgor pressure, inducing necrosis [10]. A probable explanation is that the singlet oxygen and/or other ROS produced by the PSs which are interacting with the membranes are involved in photoperoxidation, which is responsible for the membrane damage, highlighting the paired selectivity (light and PS cellular localization) [11]. One of the most impressive facts is that aPDT is efficient in multidrug-resistant strains and does not encourage resistance [12]; the repeated photosensitization of surviving cells does not induce the selection of resistant microbial strains and does not modify their sensitivity to antibiotic treatment [13,14,15].

Recently, aPDT was applied in endodontics by our team with a focus on a semi-synthetic photosensitizer obtained from the cyanobacteria *Spirulina maxima alga* (i.e., a modified chlorophyll Zn(II)chlorin *e*_6_ methyl ester [(Zn(II)*e*_6_Me]), with promising outcomes [10,16]. However, due to the disparity of root canal anatomies, as well as to the lower oxygen availability inside the root canal, researchers have been testing numerous approaches and methods to improve aPDT efficacy [17].

The present critical review implements a two-step process: (1) a concise compilation of the available evidence on PSs, as confirmed in numerous studies from diverse areas, combined with several antimicrobial strategies; and (2) analogous emerging options for the optimization of their properties and effects that might be translational and useful in the near future in endodontic research. Special focus is given to PS type, light wavelength, irradiance, and how the final antimicrobial outcomes are affected.

## 2. Bacteriophages

Filamentous bacteriophages (also recognized as phage) is a class of viruses that particularly infect Gram-negative bacteria [18]. Bacteriophages are a biological nanowire with artificially modifiable supramacromolecule (~900 nm long and ~8 nm wide), where the core is comprised of DNA and is surrounded by a cylinder of coat proteins [19]. Since coat proteins are encoded by the DNA within the phage, the phage surface can be genetically customized by fusing an external peptide to the N-terminal end (the solvent exposed end) of the coat proteins.

Phages have been used in multiple applications ranging from templating materials synthesis, biological sensing, promoting stem cell differentiation, and therapeutic delivery. Gandra and colleagues tested the application of phages in selective cancer cell killing mediated by a PDT field [18] as well as in fungus inactivation [19]. 

**Figure 2:**
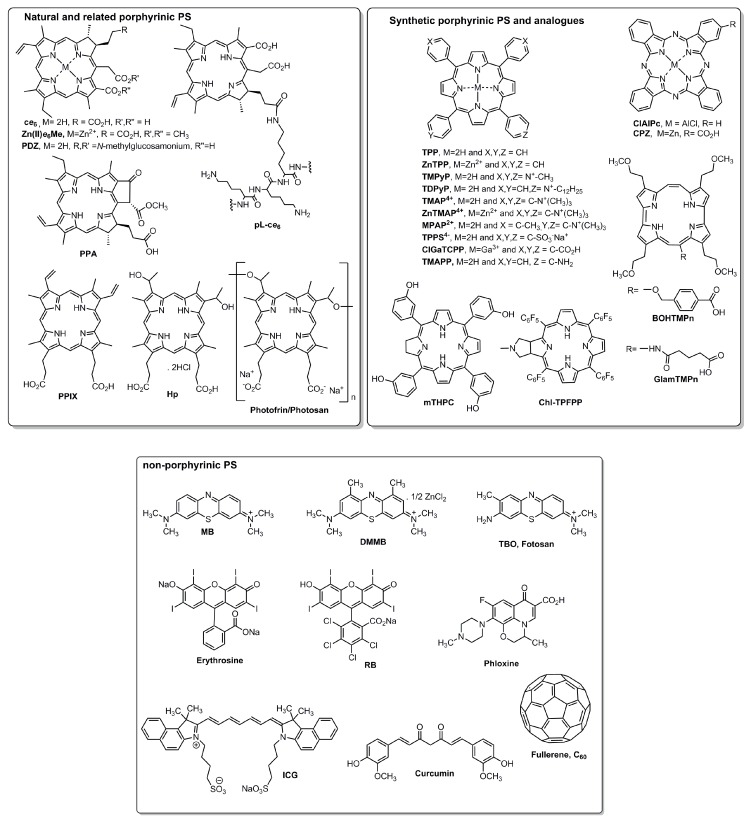
Structures of the PSs in Tables 1–6.

In 2018, Dong et al. tested the antimicrobial efficacy of the pheophorbide a (PPA, Figure 2)—a chlorophyll-based second-generation photosensitizer (PS)—after being conjugated with scFv-phage (JM) that specifically recognized the mannoprotein MP65 of the yeast Candida albicans (Table 1). The access to the conjugate PPA–JM-phage involved the cross-linking between the JM-phage N-terminus surface and the PPA acid group, mediated by the coupling agents 1-ethyl-3-(3-dimethylaminopropyl) carbodiimide–*N*-hydroxysuccinimide (EDC–NHS). It was highlighted that two treatment modalities were combined in the obtained bi-functional conjugate: aPDT by PPA targeting *C. albicans* by phage and antibody-based therapy. The final complex, PPA–JM-phage, at 5 μM was tested in *C. albicans* cells, triggered by a laser source of 658 nm for 10 min and a power intensity of 50 mW·cm^−2^. Final antimicrobial outcomes revealed that *C. albicans* cells treated with PPA–JM-phage showed a reduction of 0.92 log CFU/mL. This measurable therapeutic effect of PPA–JM-phage on *C. albicans* was observed with yeast surface morphology damage and triggered apoptosis in a metacaspase-dependent pathway. This study reveals that phage–PS conjugates may improve the therapeutic capabilities of the newly synthesized bifunctional antifungal drugs in nanophototherapeutics [19].

## 3. Drug Delivery Systems

Several carriers composed primarily of polymers and biopolymers and their associated therapeutics can be used as drug delivery systems (DDSs) to provide higher drug efficacy with reduced toxicity [20]. DDSs are designed to alter drug pharmacokinetics and biodistribution or to function as drug reservoirs, as sustained release systems [21]. The basis for controlled release formulations was introduced in 1952, and they were called the first generation; the second generation occurred between 1980 and 2010 and were called “smart delivery systems”. Since 2010, DDSs remain in a third generation referred to as modulated delivery systems, and this approach will likely persist until 2040 [22].

**Figure 3:**
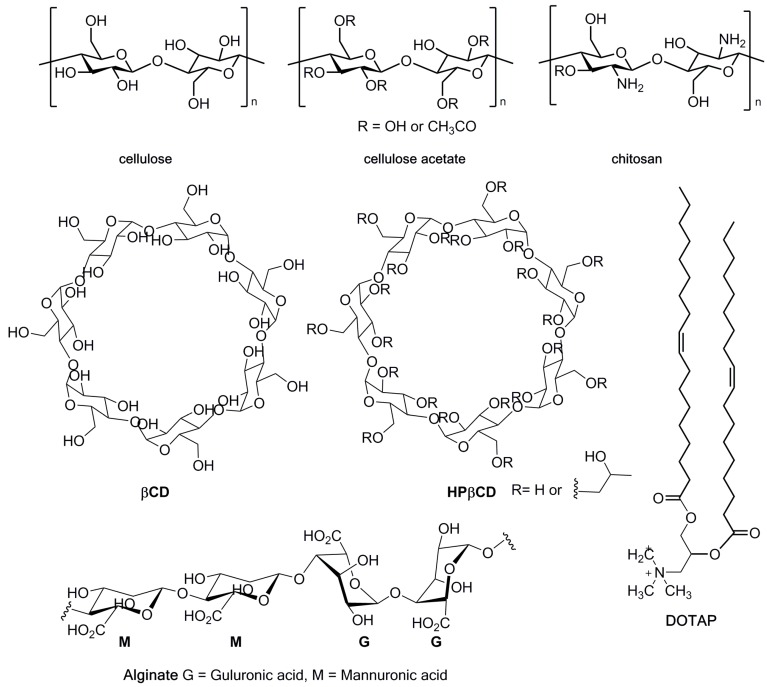
Structures of some of drug delivery systems in Tables 1–3.

DDS versatility is well-known. Here we describe the inclusion of biopolymers such as chitosan and other cellulose derivatives, alginate foam, cyclodextrins, electrolyzed water, hydrogels, and oil-based emulsions (Figure 3).

### 3.1. Cellulose

Cellulose is the most abundant biopolymer in nature, and is renewable, non-toxic, colorless, and odorless with high molecular weight, consisting of D-glucopyranose units linked through β(1–4) linkages with the empirical formula [C_6_H_10_O_5_]_n_ [20] (Figure 3). Cellulose has several advantageous properties (i.e., hydrophilicity, biocompatibility, biodegradability, and mechanical robustness) which account for its use in a vast array of fields [23]. As cellulose has a carbohydrate nature, it has inherent compatibility with biological tissues. Consequently, in 2006, Decraene et al. used cellulose acetate, a cellulose derivative (Figure 3), enclosing rose bengal (RB, λ = 549 nm) and toluidine blue-O (TBO, λ = 632 nm) to investigate the ability of such coatings to kill a range of microbes under conditions likely to be present in hospitals. For this, a white fluorescent lamp (with two prominent peaks of λ = 545 and 610 nm) was used to eradicate *Escherichia coli*, *Clostridium difficile*, a bacteriophage, the yeast *Candida albicans*, and a methicillin-resistant strain of *Staphylococcus aureus* (MRSA). The photoactive coatings were prepared by dissolving cellulose acetate in acetone, and both PSs were added to give a final concentration of 25 μM. This study revealed that PSs can retain their antimicrobial properties when embedded in the biopolymer, since the levels of killing achieved (up to a 6.7 log reduction) should be more than sufficient for surface disinfection, since microbial densities encountered on hospital surfaces are generally much lower [24] (Table 1).

Cellulose is a model support for the immobilization of bioactive molecules and within this, Rahimi and collaborators conducted an in vitro study of the photobactericidal effect of cellulosic fiber fabrics impregnated with the porphyrinic PS 5,10,15,20-tetrakis(4-*N,N,N*-trimethylammoniumphenyl)porphyrin (TMAP^4+^) and its zinc(II) ion complex (ZnTMAP^4+^) (Figure 2). The photoactive cellulosic fabrics were generated by soaking a pure cellulosic fabric pretreated with a sodium carbonate solution at 50 °C for 30 min with the PSs dissolved in phosphate-buffered saline (PBS). Subsequently, the unbound PS was completely washed. The pretreatment with the basic solution was essential to activate the cellulose hydroxyl group in order to promote electrostatic interactions with the cationic porphyrin. The photobactericidal effect was evaluated by incubating the bacteria suspension (Gram-positive *S. aureus* and Gram-negative *E. coli* and *Pseudomonas aeruginosa*) with both photoactive cellulosic fabrics in the dark for 20 min and then irradiating with a 100-W tungsten lamp at an irradiance of ~0.36 mW cm^−2^ for 30, 60, and 90 min. Although the Gram-negative bacteria were less susceptible than the Gram-positive bacterium, the photoactive material based on ZnTMAP^4+^ irradiated for 90 min exhibited 100% photoinactivation against *P. aeruginosa*, highlighting the role of the zinc atom’s presence in a synergistic inhibition of bacterial growth [25].

### 3.2. Chitosan

Chitosan (CS) is a natural structural element of fungal cells walls and crustacean exoskeletons, usually obtained by chitin acid hydrolysis (Figure 3). This polymer is biocompatible, biodegradable, and displays an attractive broad-range antimicrobial activity due to the large number of hydroxyl and free amino groups [36]. Several chemical modifications of protonated amino groups have been made on chitosan for its combination with PSs [37]. Based on this, Shrestha and Kishen tested the antimicrobial efficacy of a conjugate of rose bengal (RB, λ = 549 nm) with chitosan (RBCS, λ = 565 nm) at a concentration of 0.3 mg·mL^−1^. The RBCS was obtained from the reaction of RB with CS in the presence of the coupling agent 1-ethyl-3-(3-dimethyl aminopropyl)-carbodiimide (EDC). The biological assays involved the blend RBCS and also the non-immobilized RB and methylene blue (MB, λ = 660 nm). For this, all PSs were previously incubated in the dark (15 min) and tested on planktonic and seven-day-old biofilms of *E. faecalis* and *P. aeruginosa.* Then, the samples were irradiated by a 540-nm green light at a total light dose of 40 J·cm^−2^. Final outcomes revealed that RBCS was able to improve aPDT efficacy (>7 log reductions for biofilms; >3 log reductions for planktonic cells), highlighting the better RBCS adherence to bacteria cells and to biofilm extracellular polymeric substances (EPSs); this feature was responsible for a higher uptake into the biofilms [38] (Table 1). 

Further research was conducted by this group [26], and in 2014 they developed nanoparticles (NPs) based on cationic polymeric chitosan (CS_NPs_) covalently linked to RB (CS_NPs_RB). The NPs were prepared by adding sodium hydroxide to chitosan dissolved in acetic acid in order to increase the pH to 5, and then sodium tripolyphosphate was added under stirring. The NPs were isolated after centrifugation, and their conjugation to the RB acid group was performed in the presence of EDC–NHS leading to CS_NPs_RB. The antimicrobial efficacy of CS_NPs_RB was tested in *E. faecalis* biofilms with root canal tissue inhibitors such as dentin, dentin matrix, pulp remnants, bacterial lipopolysaccharides, and bovine serum albumin (BSA). PSs were incubated in the dark, period recognized as pre-incubation time (PIT) for 15 min and irradiated with a lamp fitted with 540-nm filtered fiber for 1.66 and 3.33 min (total light dose of 5 and 10 J·cm^−2^). The study showed that the CS_NPs_RB was less toxic to fibroblasts than RB, and the higher affinity of CS_NPs_RB for bacterial cell surfaces and the singlet oxygen release after photoactivation of RB provided a synergistic mechanism for CS_NPs_RB to exert antibacterial efficacy even in the presence of tissue inhibitors (reduction > 8 log) acting as a novel antibacterial agent with distinct and potential benefits in root canal disinfection [26] (Table 3).

### 3.3. Alginate Foam

Alginate is a natural anionic biomaterial of polysaccharide polymers extracted from brown algae (Phaeophyceae), and has been described as the main component of alginate foams [39]. Alginate is a set of linear copolymers with blocks of (1,4)-linked β-d-mannuronate (M) and α-l-guluronate (G) residues and differ in each block length, likewise M and G contents, Figure 3. 

Cyclodextrins (CDs) are macrocyclic cone-shaped oligosaccharides with a hydrophilic outer surface and a lipophilic internal space composed of glucopyranose units linked by glycosidic oxygens. This linkage and hydrogen bonding contribution create a rigid hydrophobic cavity that can carry a variety of molecules of suitable size [40]. CDs are large molecules with hydrogen donors and acceptors that are able to permeate through biological membranes without disrupting the lipid layers of the barrier, delivering insoluble hydrophobic drugs by making the drug available at the surface of the biological barrier, but CDs do not invade lipophilic membranes. Hydrophilic CDs increase the delivery of small lipophilic molecules when the permeation through an unstirred water layer is the rate-limiting step of permeation. Moreover, CDs in excess may decrease the drug availability. Three available CDs are named α-, β-, and γ-CDs (with 6, 7, or 8 glucose units, respectively), and differ in their ring size and solubility [40]. Hegge et al. tested two derivatives named hydroxypropyl-β-cyclodextrins (HPβCDs, Figure 3), and hydroxypropyl-γ-cyclodextrins (HPγCDs) as curcumin solubilizers in several alginate foams as a DDS for aPDT in infected wounds mediated by curcumin (Figure 2). By selecting β- and γ-CDs, curcumin was uniformly distributed throughout the hydrophilic foam matrix and released in the monomeric form. Several assays indicate that the HPβCD–alginate foam was the best excipient, since it stabilized curcumin against photodegradation, developing a solubilizing effect as a promising DDS for aPDT [27] (Table 1). 

One year later, with the same investigation group, Hegge et al. affirmed that some alginate foams remained intact after hydration and would be possible to remove from the wound prior to irradiation without instigating tissue damage, since curcumin has an extremely low solubility in water and may aggregate in aqueous environment [28]. Subsequently, CDs and polyethylene glycol 400 (PEG 400) were selected as curcumin solubilizers to provide a burst sonosensitizer (SS) release. Exposure to the prepared foams in combination with visible light irradiation resulted in >6 log reduction of *Enterococcus faecalis* cells.

### 3.4. Electrolyzed Water

Electrolyzed water is produced by the electrolysis of water containing sodium chloride (NaCl). NaCl dissolves in water and dissociates into positively (Na^+^) and negatively (Cl^−^) charged ions. Subsequently, hydroxide (OH^−^) and hydrogen (H^+^) ions are also produced. The negatively charged ions (OH^−^, Cl^−^) move toward the anode where electrons are released and hypochlorous acid, hypochlorite ion, hydrochloric acid, oxygen gas, and chlorine gas are generated. Positively charged ions (Na^+^, H^+^) move toward the cathode where they gain electrons, resulting in the generation of sodium hydroxide and hydrogen gas. Two types of EW are generated simultaneously; acidic electrolyzed water (AcEW) is formed on the anode (pH 2–3), and alkaline electrolyzed water (AlEW) is produced at the cathode (pH 10–13) [29]. EW exhibits antimicrobial activity against a variety of microorganisms including bacteria, viruses, fungi, and spores in a relatively short exposure time (within 5 to 20 s). Based on this, Ishiyama and collaborators tested the photo-irradiation activity of three xanthene compounds (RB, erythrosine, and phloxine; Figure 2) combined with AcEW or AlEW against experimental 24-h biofilms of *S. mutans*. Final outcomes revealed that only the photo-irradiated RB-AlEW showed noticeable bactericidal activity with a >3 log reduction of the viable bacterial count, as the AcEW group showed no physical damage to the extracellular matrix of the biofilm. As a consequence, it is strongly advocated that RB-AlEW could be applied to treat dental biofilms. Since Ishiyama’s study dealt with a single-species *S. mutans* biofilm, the effect on mixed-species biofilms should be examined in the near future in order to further ascertain the feasibility of the treatment’s application in dentistry [41] (Table 1).

### 3.5. Hydrogels 

Hydrogels are a group of hydrophilic polymeric materials that can hold a large amount of water in their three-dimensional networks due to their hydrophilic structures. The polymer origin may be natural or synthetic and can be used to carry agents in a targeted manner, in which the dispersion medium is water [42]. 

Macrogels can stay at the target sites for quite a long time due to their inherent low fluidity, and are commonly used for photodynamic cancer therapy, while microgels/nanogels represent an emerging material in aPDT. Moreover, hydrophobic and hydrophilic PSs can be carried in a microgel/nanogel system through physical (encapsulation) or chemical (conjugation) methods [43]. Additionally, hydrogels can be used several times at one site due to their fluidity. Microgels and nanogels have several benefits for aPDT, as the gels’ small sizes enable PSs to effectively reach the target sets, and the microgel/nanogel surface can be further modified with functional groups or targeting agents in order to alter the biological or physical properties, improving the biodistribution pharmacokinetics, cell uptake, and targeting ability [44].

McCarron’s team tested cross-linked poly (vinyl alcohol) (PVA) hydrogels as a DDS combined with two cationic PSs—MB and 5,10,15,20-tetrakis(1-methylpyridinium-4-yl)porphyrin tetra-tosylate (TMPyP, Figure 2)—for aPDT in infected wounds with both planktonic and biofilm-grown MRSA (Table 1). PVA was dissolved in deionized water and sodium tetraborate solution was added to give a final hydrogel comprising 20% *w/w* and 8% *w/w* polymer and cross-linker. Defined amounts of MB or TMPyP were added to the borax solutions prior to addition to PVA solutions in order to produce drug-loaded variants, and both were irradiated with a Paterson lamp (635 nm, 100 m·W·cm^−2^), as described in Table 1. Final outcomes revealed that TMPyP–PVA hydrogels were the best antimicrobial conjugate PS, possibly due to their cationic character relative to MB; even the presence of calf serum had no significant effect on the mean percentage kill of MRSA [30]. 

### 3.6. Lipid Delivery Systems

The increase in ^1^O_2_ production efficiency is a crucial goal to achieve a high aPDT efficacy. Indeed, the hydrophobic nature of most PSs promotes their aggregation in aqueous media, reducing the aPDT efficacy with a minor singlet-oxygen formation by a self-quenching effect in the excited state [45]. In order to preserve PSs’ monomeric state, and to increase aPDT efficacy, several transporters and delivery strategies (e.g., lipid delivery systems, LDSs) have recently been developed as an encapsulation technique to also protect the host from the PS cytotoxicity. 

LDSs provide several advantages, including loading capacity, biodegradability, and biocompatibility [46,47]. Moreover, LDSs have two main advantages: firstly, they can accommodate both hydrophobic and hydrophilic PSs; secondly, the synergistic outcome of positively charged and highly fluid components of LDS increases the uptake of PSs by microorganisms, as well as the overall phototoxicity [48]. In this subchapter, LDSs are subdivided by alphabetical order into invasomes, liposomes (the classical vehicle), and micelles (Table 1). 

#### 3.6.1. Invasomes

Invasomes (INVs) are liposomal vesicles of phosphatidylcholine embodying small amounts of ethanol and terpene mixtures used as potential carriers with enhanced penetration compared to the conventional liposomes (LIPs). Ossmann et al. evaluated the photodynamic killing efficacy of 5,10,15,20-tetrakis(*m*-hydroxyphenyl)chlorin (mTHPC; 24 h dark incubation) at a concentration of 50 μM incorporated in LIPs and INVs in dentinal tubules with *E. faecalis* 48-h biofilms [31] (Table 1). The INV–mTHPC manufacturing process was previously described [32], and for the LIP–mTHPC conjugate group, mTHPC was incorporated into a lipid double membrane consisting of dipalmitoyl-phosphatidyl choline and dipalmitoyl-phosphatidyl glycerol (Foslipos, Biolitec AG, Jena, Germany). The lipid envelope of INV contains soybean phosphatidyl (Phospholipid GmbH, Cologne, Germany), 10% ethanol, and 1% of a terpene mix (d-limonene, citral, 1,8-cineole). Both conjugates were added to roots, and a laser (λ = 652 nm; 0.25 W, 100 J cm^−2^) was applied for 452 s. aPDT treatment with mTHPC–INV and mTHPC–LIP resulted in significant bacterial reduction, since both were capable of efficiently suppressing *E. faecalis* inside dentinal tubules ≥ 300 μm (it was assumed that in vivo *E. faecalis* may colonize dentinal tubules to depths of 150 μm or more). Moreover, these outcomes were more effective than temporary dressing with 1% of chlorhexidine gel (positive control). Still, the antimicrobial efficacy of mTHPC–INV tended to be higher than that of mTHPC–LIP, mainly based on the high deformability of the lipid cover, the terpene content, ethanol, as well as the presence of a strong osmotic gradient [40].

#### 3.6.2. Liposomes 

Liposomes (phospholipids only) are spherical vesicles with at least one lipid bilayer, can vary in size on the order of micrometers, and have been widely investigated due to their lipidic structure’s similarity with human cell membranes. However, phospholipids’ high costs and instability are massive disadvantages. In this approach, the most difficult step is the encapsulation of molecules in the LIP, since the preparation is a fastidious process based on bench experience. Previously to Ossmann and co-workers [31], Ferro et al. tested a novel and positively charged *meso*-substituted porphyrin, namely 5-(1-dodecanoylpyridinium-4yl)-10,15,20-triphenylporphyrin (TDPyP, Figure 2), incorporated into a poly-cationic LIP named as *N*-[1-(2,3-dioleoyloxy)propyl]-*N*,*N*,*N*-trimethylammonium chloride (DOTAP, Figure 3), in the photoinactivation of MRSA (Table 1). For aPDT experiments, the conjugate TDPyP–DOTAP (0.7 μM, PIT: 2 h) was irradiated at 20 °C by a quartz-halogen lamp (5, 10, 15, 20, 25, 30 min; 50 and 100 m·W·cm^−2^) equipped with a heat-reflecting and UV-blocking filter. With TDPyP–DOTAP, an effectively 4.5 and 6 log reduction in MRSA survival was obtained, respectively, upon 5 and 10 min of light exposure [32]. 

#### 3.6.3. Micelles

The use of polymeric pH-sensitive micelles (MICs) is a promising LDS of several molecules such as PSs. MICs are described as surfactant monolayers, and when compared to LIPs, MIC preparation can be much less expensive and simpler, since MIC scale-up is not difficult [49].

Tsai et al. tested Hp (hematoporphyrin dihydrochloride, Figure 2) condensed in LIPs (Hp–LIP) and MICs (Hp–MIC) using a commercially available poloxamer Pluronic F127 (PF127) to solubilize and encapsulate Hp against Gram-positive bacteria (Table 1). For this investigation, Hp–LIP was obtained from a modified reverse-phase evaporation and extrusion method. Hp–MIC was obtained by a reverse-phase evaporation method. Both conjugates were incubated for 30 min in the dark and irradiated with a high-power LED (λ = 635 nm; 60 m·W·cm^−2^). Remarkable aPDT outcomes were achieved by the application of 0.25 μM Hp–MIC against *S. aureus*, with complete bacteria elimination (similar patterns were found in *S. epidermidis* and *S. pyogenes*). For Hp–LIP, a higher concentration (0.5 μM) was needed to achieve the same result. To sum up, with the lowest Hp concentration, the conjugate Hp–MIC had a complete bactericidal effect and it was hypothesized that the smaller size of Hp–MIC may be a tremendous advantage. Final results indicated that Hp–MIC exerted better aPDT efficacy than Hp–LIP, showing that this cheaper formulation may be useful for the treatment of local infections in the future. Additionally, Tsai et al. solubilized and encapsulated chlorin *e*_6_ in MIC (*e*_6_–MIC) and found an aPDT efficacy increase while avoiding PS aggregation, improving the PS’ potential [33].

### 3.7. Oil-Based Emulsions

Oil-based emulsions (ObEs) have recognized antimicrobial properties due to their hydrophobic partition in the phospholipid bilayer of microorganisms’ membranes, promoting cell wall porousness, and are apportioned into microemulsions (MEs) and nanoemulsions (NEs) [50]. MEs are thermodynamically stable colloidal dispersions consisting of small spheroid particles (comprised of oil, surfactant, and possibly co-surfactant) dispersed within an aqueous medium. In contrast, NEs are thermodynamically unstable colloidal dispersions consisting of two immiscible liquids, with one of the liquids being dispersed as small spherical droplets (*r* < 100 nm) in the other liquid [51]. 

In 2016, Rout et al. tested the effectiveness of a loaded ME–TBO in *P. aeruginosa* inhibition compared with TBO (dispersed in water) or with ME–TBO and ethylenediaminetetraacetic acid (EDTA) at several concentrations (Table 1). Photoinactivation studies were performed for 15 min using an LED system centered at λ = 600 nm with a total light dose of 0.61 J·cm^−2^. Final results revealed that ME–TBO (5 μg·mL^−1^) significantly inhibited *P. aeruginosa* (about 2 log bacterial reduction), and aPDT efficiency using TBO was dosage dependent. Moreover, ME–TBO–EDTA promoted a 3 log bacterial reduction. This efficiency improvement could be due to EDTA’s chelating properties towards microorganisms’ divalent cations (Ca^2+^, Mg^2+^), disseminating bacterial cell structure instability and consequently reducing the electrostatic repulsion posed to the cationic TBO. Therefore, it is hypothesized that a better penetration is achieved with EDTA, enabling lower dosages of TBO to be used, avoiding dark toxicity [34].

Under the context of NE, Ribeiro et al. tested the photoinactivation ability of chloro-aluminum phthalocyanine (ClAlPc) encapsulated in cationic and anionic NEs against methicillin-susceptible *S. aureus* and MRSA in planktonic and biofilm forms (Table 1). As a main conclusion, with the final concentration of 31.8 μM, the cationic NE–ClAlPc irradiated with red light (λ = 660 nm, total light dose of 50 J·cm^−2^) for 26 min promoted similar results in reducing methicillin-susceptible *S. aureus* and MRSA planktonic suspensions. Afterwards, cationic ClAlPc efficiency was dependent on the DDS, superficial load, and light dose, since the anionic NE–ClAlPc was not capable of reducing MRSA biofilm metabolism [35].

## 4. Metal–Organic Frameworks

Metal–organic frameworks (MOFs) are permanently microporous hybrid materials constructed via strong metal–ligand covalent bonds between inorganic clusters and organic linkers in one single material with structural molecular designability with chemical functionalizability and tunable porosity [52]. MOFs have crystalline structures and are typically characterized by large internal surface areas and uniform but tunable cavities, and these characteristics make MOFs promising for a variety of applications, including PS delivery. MOFs have emerged as a promising new class of porous materials with some authors including them incorrectly in the nanoparticles field (MOFs can incorporate nanoparticles and/or be designed in nanoscale) [53].

Recently, Golmohamadpour et al. assessed the aPDT efficacy of three indocyanine green (ICG)-loaded MOFs against *E. faecalis* 24-h biofilms in human mandibular first premolars as the first report of ICG–MOF for aPDT in endodontic infections [54] (Table 2). For this, authors synthesized three different MOFs from the Materials Institute Lavoisier (MIL) family reinvigorated by its considerable loading capacity: Fe-MIL-88B-NH_2_ (Fe-88); Al-MIL-101-NH_2_ (Al-101); and Fe-MIL-101-NH_2_ (Fe-101). Subsequently, they investigated the in vitro antimicrobial activity of indocyanine green loaded with the three MOFs (ICG-Fe-88, ICG-Fe-101, ICG-Al-101). For aPDT tests, all PS conjugates were prepared at 100 μg·mL^−1^ and triggered by a diode laser (λ = 810 nm; 250 mW; 31.2 J·cm^−2^; 60 s). The antimicrobial outcomes after aPDT assays revealed that the ability to reduce the colony count of *E. faecalis* was significantly improved by the conjugates up to 45.12% (ICG-Fe-88), 60.72% (ICG-Al-101), and 62.67% (ICG-Fe-101). Moreover, the gene expression of *E. faecalis* as one of the reasons for plaque formation on teeth was considerably decreased in the presence of ICG-Fe101-after PDT treatment (hypothesized to be due to iron activity by itself), since this conjugate was the highest spacious carrier. Final outcomes revealed ICG-Fe101 as a promising PS due their structural characteristics (strong stability and great carrier) as well as the antimicrobial activity of iron metal by itself [54].

## 5. Nanoparticles

Nanotechnology allows delivery vehicle development to overcome physiologically imposed barriers, enabling new approaches for reducing the unwanted systemic side effects of PSs, increasing PS target efficiency, and amplifying aPDT outcomes. The advantages of nanoparticles (NPs) depend on their properties, which are size and surface dependent. Moreover, NPs’ most important feature is their large external surface area as well as their high surface-to-volume ratio, which dominates NPs’ physicochemical properties. NPs start to become significant at a length scale below 100 nm, which defines the arbitrary but scientifically accepted definition of nanomaterials and NPs in particular [65]. Allaker and Memarzadeh outline the most-used NPs to combat oral infections and subdivide them in four groups; (i) antimicrobial NPs for oral biofilm removal (silver, copper, gold); (ii) metal oxide nanoparticulates (copper oxide, zinc oxide, titanium dioxide); (iii) anti-adhesive NPs (silica, chitosan, hydroxyapatite, and calcium-phosphate-based systems; and finally (iv) NPs incorporated in polymeric materials to prevent aggregation [65], because in humans NPs with diameter ≤ 100 μm have a high likelihood of aggregating and with diameters ≥ 5 μm may embolize vessels. 

Samiei et al. performed a systematic review of in vitro studies to obtain an antimicrobial effect from NPs in endodontics, and concluded that silver NPs were the most-studied agent for their antimicrobial behavior in root canal infections [66]. 

In 2015, Lucky et al. classified NPs utilized in PDT into three groups. In the first group, PS carriers, where NPs act as delivery systems. In the second group, NPs act as PSs in themselves, because certain nanoscale materials have the ability to generate ROS due to their unique optical absorption properties (e.g., fullerenes, titanium-dioxide, and zinc-oxide NPs). In the third group NPs act as energy transducers of PSs in which several NPs not only transport PSs, but also dynamically participate in energy transfer to the attached PS [67]. 

In the present study, the authors overview essential information on several NPs combined with several PSs to improve PDT antimicrobial efficacy with no intention of focusing on anti-tumoral PDT (Table 2).

### 5.1. Carbon

Carbon nanomaterials have been intensely tested in several forms (e.g., carbon nanotubes, graphene, and fullerenes) as promising adjuvants of antimicrobial PSs due to carbon’s antimicrobial properties and antiseptic behavior. 

#### 5.1.1. Carbon Nanotubes

Carbon nanotubes (CNTs) can be arranged in single-walled (SWCNTs) and multi-walled (MWCNTs) structures. Both have exclusive properties which distinguish them from other conventional materials (i.e., distinctive tubular arrangement, large modifiable surface, modifiable sidewall, outstanding conductivity, tensile strength, and strong stability) [68], while possessing distinct biocompatibility. Focused on the importance of CNTs diameter, Kang et al. compared the antimicrobial activity of highly purified single- with multi-walled CNTs in *E. coli* inactivation, and observed that the antimicrobial activity of SWCNTs was higher than that of MWCNTs [69]. Recently, Sah et al. synthetized a nano-composite of an amine-functionalized porphyrin conjugated with SWCNTs and tested it in *S. aureus* planktonic suspensions to observe its antimicrobial activity (Table 2). For this, SWCNTs were combined with 5,10,15-triphenyl-20-(4-aminophenyl)porphyrin (TMAPP) and irradiated by a halogen-tungsten lamp (λ = 419 nm; 500 W; 10 min). Final outcomes showed that with a minimum irradiation period of 10 min, the conjugate TMAPP–SWCNTs showed antimicrobial properties not only against *S. aureus* dispersed in aqueous solutions, but also on infected nitrocellulose membranes [55]. 

In another study, Banerjee et al. tested the mechanical strength of MWCNTs as a scaffold to embody protoporphyrin IX (PPIX) and concluded that PPIX–MWNTs conjugates could effectively disable *S. aureus* when irradiated with visible light for 15 min [56] (Table 2). 

#### 5.1.2. Nano-Graphene Oxide

Graphene is the simplest carbon nanostructure form, and nano-graphene oxide (NGO) is a water-soluble derivative with excellent biocompatibility and a wide field of applications. NGO has the ability to load huge amounts of hydrophobic PSs, since it is able to solubilize hydrophobic dyes [70]. The effect of indocyanine green (ICG) loaded on a novel nano-graphene oxide was evaluated for effective aPDT against *E. faecalis* 24-h biofilms in root canal disinfection (Table 2). In this study, ICG’s photodynamic properties were improved through ICG’s incorporation into NGO (ICG–NGO), and when irradiated for 60 s (λ = 810 nm; 50 mW·cm^−2^; 31.2 J·cm^−2^) at 200 μg/mL, this new PS conjugate showed a significant reduction in *E. faecalis* biofilms of up to 99.4%. The anti-biofilm potential of NGO–ICG after light irradiation was 1.3 times higher than that of non-immobilized ICG (1000 μg·mL^−1^). The use of this new NIR photo-triggered drug delivery system has several advantages, such as its antimicrobial and anti-biofilm properties, cost-effectiveness, lower toxicity, and reduced teeth discoloration, and the authors recommend it as an adjuvant to conventional irrigation for endodontic treatment [57]. 

#### 5.1.3. Fullerenes 

Fullerenes are allotropic carbon forms (the third most stable after diamond and graphite)—a class of closed-cage nanomaterials made exclusively from carbon atoms such as C_60_, C_70_, and C_84_ (Figure 2). When conjugated with functional groups, fullerenes become soluble and can act as PSs that are particularly effective at mediating Type 1 photochemical mechanisms, contrary to the Type 2 generation of singlet oxygen that dominates other PSs [58,71]. Fullerenes have condensed aromatic rings present in the compound, leading to an extended π-conjugation of molecular orbitals, and with their large number of conjugated double bonds efficiently absorb light in the UV and visible spectral regions and have a high triplet yield that can generate ROS upon illumination [71]. In contrast to pristine fullerenes (which are highly hydrophobic and insoluble in aqueous media), closed-cage fullerenes can be derivatized by the attachment of organic ligands containing suitable functional groups to provide water solubility and to allow the fullerenes to recognize and bind to biological targets such as bacterial cells, making them largely suitable for biological applications [71]. 

Tegos and his team were the first to demonstrate that soluble functionalized fullerenes with one, two, or three quaternary pyrrolidinium groups (1 and 10 μM) after a short incubation time (10 min) followed by illumination with white light (400–700 nm; 200 mW·cm^−2^; 16 J·cm^−2^), in direct comparison with TBO, were efficient PSs by themselves against Gram-positive and Gram-negative bacteria as well as fungi (4–6 log reductions) [58] (Table 2). Moreover, Zhang et al. studied C_60_ fullerene bearing a decaquaternary chain and/or a decatertiary amino group as PS. From these studies performed in the presence or absence of potassium iodide (KI), the main conclusion reached was that 200 μM of C_60_ fullerene bearing a decaquaternary chain and a decatertiary amino group combined with KI was able to eradicate both bacteria types and fungi (Table 2). All specimens were excited with white light (100 mW·cm^−2^; 120 J·cm^−2^) and ultraviolet A (UVA; 20 mW·cm^−2^; 20 J·cm^−2^), and UVA was five times more effective than white light and KI addition increased the light-mediated killing [59].

### 5.2. Gold 

Gold (Au) can give rise to different forms of nanoparticles (e.g., colloidal nanoparticles, nanorods, nanospheres, nanostars, nanoshells, nanobelts, nanoclusters, nanocages, nanoprisms, nanostars, branched NPs, etc.). In endodontics, Pagonis et al. studied the in vitro antimicrobial effect of colloidal Au nanoparticles complexed with poly (lactic-*co*-glycolic acid) (PLGA) and MB, resulting in a new PS complex named as PLGA–Au–Pluronic–MB. For this, PLGA–Au–Pluronic–MB (6.25 μg·mL^−1^; PIT: 10 min) in suspension was added to *E. faecalis* planktonic state and in root canals infected with 3-day biofilms (Table 2). The conjugate was triggered by a red diode laser (λ = 665 nm; 1 W; 30–60 J·cm^−2^) and since antimicrobial outcomes were lower than 3 log reductions, the new conjugate is suggested as an adjunct in antimicrobial endodontic treatment in biofilms [60]. 

### 5.3. Platinum

Platinum (Pt) is known to inactivate microbes by interacting with their enzymes, proteins, or DNA, and to restrain cell proliferation or cell division, since it possesses the ability to enter cells [72]. Thus, in 2014, Managa et al. studied the antimicrobial efficacy of hexagonal PtNPs conjugated to the gallium(III) complex of 5,10,15,20-tetrakis(4-carboxyphenyl)porphyrin (ClGaTCPP). The aPDT efficacy of the ClGaTCPP–PtNPs conjugate was tested in several concentrations (0.025, 0.05, 0.1, 0.25 mg·mL^−1^) and embedded in electrospun polystyrene fiber against *S. aureus*. The photodynamic assays were performed with a general electric quartz line lamp (300 W; 0.05 W·cm^−2^; 0.8 J·cm^−2^) using different irradiation times (ITs) of 0, 30, 60, and 90 min (Table 2). Final outcomes revealed that ClGaTCPP–PtNPs at 0.2 mg·mL^−1^ impregnated in a electrospun fiber was able to give a significant bacterial inhibition [61].

### 5.4. Silica

Silica (SiO_2_)-based NPs are transparent, usually do not alter the PSs’ spectral characteristics, and are usually considered to be good carriers for PS delivery (they are water dispersible, chemically and photodynamically stable, and their surface can be easily modified). Guo et al. manufactured a new PS made from SiO_2_ combined with RB (SiO_2_–RB) that was found to be highly efficient in the inactivation of Gram-positive bacteria (MRSA and *S. epidermis*, Table 2). For this, the silica surface was functionalized with amine groups (NH_2_), creating the complex SiO_2_–NH_2_–RB as a PS. In this study, authors used the SiO_2_–NH_2_–RB complex (6 mg·mL^−1^; PIT: 30 min) irradiated with a light source (λ = 525 nm; 14 mW·cm^−2^; 33 J·cm^−2^; IT: 40 min) and found that SiO_2_–NH_2_–RB was more powerful than RB alone, with a killing efficiency of 2 log reduction in the viability count. Additionally, the SiO_2_–NH_2_–RB quantum yield of generating ^1^O_2_ was lower than that of RB. The amount of RB in the SiO_2_–NH_2_–RB used in the experiments was approximately half that in free RB solution, and authors hypothesized that this could be due to the higher localization of RB to the cell surface when attached to the SiO_2_ NPs [62].

### 5.5. Silver

Among metal NPs, silver nanoparticles (AgNPs) exhibit the most pronounced antimicrobial effect, with bacterial cell-wall destruction and subsequent disruption of intracellular balance, with recognized better effectiveness against Gram-negative than Gram-positive bacteria [73]. Additionally, AgNPs can be combined with several agents, such as antibiotics, dyes, or other molecules to extend their antimicrobial outcomes. In aPDT, Lyutakov et al. tested polymethylmethacrylate (PMMA) doped with a porphyrin and AgNPs as a light-activated antimicrobial material (Table 2). The selected porphyrin, 5,10,15,20-tetraphenylporphyrin (TPP), was attached to films based on PMMA conjugated with AgNPs (TPP–Ag–PMMA) as a new PS against *S. aureus* and *P. aeruginosa* bacteria. Concentrations of Ag and TPP in the dried polymer film were 10% and 5% respectively. The weak antimicrobial properties of the TPP–Ag–PMMA against *P. aeruginosa* (without irradiation) were enhanced after blue light activation (λ = 405 nm; 110 mW). Moreover, it was evident that TPP–Ag–PMMA under light activation (3 h) led to the complete destruction of *S. aureus*, but only to partial destruction of *P. aeruginosa* [64]. 

### 5.6. Superparamagnetic Iron Oxide Nanoparticles

Superparamagnetic iron oxide nanoparticles (SPIONs) have several advantages, as they can be used in magnetic resonance imaging, cell labeling, tissue targeting, hyperthermia, and drug delivery [74]. In nature, SPIONs are frequently found in different forms, such as hematite (α-Fe_2_O_3_), maghemite (γ-Fe_2_O_3_), and magnetite (Fe_3_O_4_), among others. The most-studied SPIONs are hematite and magnetite [75]. As SPIONs have a certain propensity to agglomerate, their surfaces can be functionalized with hydrophilic ligands (e.g., polyethylene glycol, dextran, phospholipids, and chitosan), and they can then be used in combination with PSs such as chlorin *e*_6_ and hematoporphyrin to form water-stable SPIONs for aPDT (Figure 2). With magnetic manipulation, SPIONs–PSs with iron oxide cores can be directed to a location site and with light source activation can promote antimicrobial effects in microbial cells with lower host side effects. Based on this, Thandu et al. investigated a porphyrin–SPION nanoconjugate derived from TPP and magnetite (TPP–Fe_3_O_4_) as an antimicrobial magnetic PS against bacteria (Table 2). Their main goal was to combine the PS photoactivity with the possibility of recovering the PSs with magnets, since after treating skin and oral infections the PSs cannot be left unattended after the depletion of microorganisms. When irradiated with a blue light (λ = 470 nm; 48 W·m^−2^) for 180 min, TPP–Fe_3_O_4_ (0.5 μM; PIT: 10 min) promoted a suitable inactivation of Gram-positive bacteria (with better antimicrobial outcomes for *S. aureus*), but failed in Gram-negative bacteria eradication [64].

## 6. Efflux Pump Inhibitors

Depending on the specific class they belong to, efflux pump inhibitors (EPIs) are either single- or multiple-component systems containing an inner membrane transporter and an outer membrane channel with a periplasmic adaptor protein. Moreover, EPIs have become broadly recognized as major components of the antimicrobial resistance of several classes of antibiotic [76]. EPIs can be divided into five families: a major facilitator superfamily (MFS), ATP (adenosine triphosphate)-binding cassette (ABC), resistance-nodulation-division (RND), the small multidrug resistance (SMR) and the multidrug and toxic compound extrusion (MATE). Except for the RND superfamily, which is only found in Gram-negative bacteria (but not exclusive to them), EPIs of the other four families (i.e., MFS, ABC, SMR, and MATE) are widely distributed in both Gram-positive and Gram-negative bacteria [77].

Tegos and co-workers point out that efflux pump inhibitor use could enhance aPDT efficiency, suggesting that PDT is hindered by the penetration of the drug into the bacterial cell [78,79]. In 2006, Tegos and Hamblin tested if the amphipathic cationic phenothiazinium salts TBO, MB, and 1,9-dimethylmethylene blue (DMMB, λ = 635 nm) could be substrates of microbial multidrug resistance (MDR) pumps (Figure 2). Based on this, they used established MDR-deficient mutants of *S. aureus* (NorA), *E. coli* (TolC), and *P. aeruginosa* (MexAB) and found 2–4 logs more killing than seen with wild-type strains by phenothiazinium PSs in opposition to non-phenothiazinium-based PSs such as poly-*l*-lysine–chlorin *e*_6_ conjugate (pL-c*e*_6_, λ = 660 nm) and RB (Table 3). Within this, specific MDR inhibitors might be used in combination with phenothiazinium PSs to enhance their photodestructive efficiency. It is also important to mention that Tegos and Hamblin raised the hypothesis of some bacteria developing resistance to phenothiazinium-based aPDT due to the selective survival of strains with increased MDR expression levels [78].

Later, from a Tegos co-worker, verapamil (hydrochloride), an FDA-approved *P*-glycoprotein efflux pump inhibitor that blocks calcium channels and inhibits the ATP (adenosine triphosphate)-binding cassette (ABC) of several bacteria species [84], was tested as a specific microbial EPI to potentiate aPDT in two strains of *E. faecalis* (OGIRF and FA 2-2) using the cationic MB and anionic RB to assess their ability to inactivate planktonic suspensions and 4-day-resident biofilm and biofilm-derived cells (Table 3). Both PSs were used at the same concentration (100 μM) and MB was combined with 100 μM of verapamil (MB–V). After 15 min of dark incubation, a non-coherent light source with interchangeable fiber bundles (λ = 540 nm for RB; 660 nm for MB) and a total power output provided from 300 to 600 mW was applied with a light dose ranging from 2 to 15 J·cm^−2^. The results obtained indicated that the aPDT susceptibility order was planktonic cells > biofilm-derived cells > biofilms. Additionally, MB produced a higher antimicrobial outcome than RB. Moreover, the MB–V complex enhanced the PS uptake by the bacteria cells, improving the aPDT efficacy to eradicate *E. faecalis* biofilm-derived cells and biofilm structures [84].

## 7. Light Delivery Systems

For PDT, the light wavelength must be selected according to the PSs’ absorption maximum in order to optimize the aPDT efficiency, and this parameter is of paramount importance. Some PSs have lost their microbial capability because they needed specific light requirements and their simultaneous combination can trigger associated costs. 

In 2015, our research group reviewed 29 articles on aPDT applied to endodontics, and concluded that in terms of commercial light sources, there were three light delivery systems that have been thoroughly studied: Denfotex (λ = 635 nm, SaveDent; Denfotex, Inverkeithing, UK), Helbo (λ = 660 nm, Helbo Photodynamic Systems, Grieskirchen, Austria), and (λ = 628 nm, FotoSan® 630 LAD pen, CMS Dental, Copenhagen, Denmark). Moreover, the irradiation time (IT) of aPDT treatment varying between 30 s and 20 min is an essential parameter to consider [12]. Furthermore, in endodontic aPDT, two other aspects have been explored but need supplementary studies. Firstly, for intracanal polymicrobial biofilms removal, is it mandatory to use a light source with an intracanal fiber? Secondly, in apical periodontitis cases, could periapical tissues be irradiated externally through the periapical bone?

### 7.1. Light with or with No Fiber

In 2011, Nunes et al. performed an ex vivo study to evaluate the aPDT effectiveness in eradicating *E. faecalis* 21-day biofilms, with or without the aid of an intracanal optical fiber, established at the working length (WL). Extracted human single-rooted teeth were pre-incubated with MB for 5 min and irradiated for two different periods (90 s and 3 min) with a diode laser (λ = 660 nm, 300 mW·cm^−2^, 8–16 J), and a flexible cylindrical intracanal optical fiber with a plain tip was tested at the established WL with spiral movements, from apical to cervical, to improve the light diffusion throughout the root canal. Final outcomes revealed that, under the conditions described, aPDT was effective against *E. faecalis* (99.64%), independent of the use of a fiber (99.65%) [80] (Table 3). The same conclusions were drawn by Garcez et al. [85] and Rödig et al. [86]. In an experimental study with infected human teeth, the relevance of fiber insertion depth inside the root canal (apical vs. coronal) was tested. For this purpose, single-rooted extracted teeth were infected with 72-h *E. faecalis* biofilms. Subsequently, low viscosity FotoSan® agent (CMS Dental, Copenhagen, Denmark) (TBO; 100 μg·mL^−1^; pre-incubation time (PIT) of 2 min) was added and irradiated for 60 s with LEDs (λ = 628 nm, 2000 mW·cm^−2^) with a fiber located at the root apical third (WL) or at coronal third (WL−5 mm). aPDT was successful in *E. faecalis* eradication, and insertion depth had a negligible influence on aPDT final outcomes (<0.5 log reductions) [86,87]. Therefore, the use of an optical fiber in direct contact with the root canal wall may not be as essential as in high-power laser therapy for bacteria. This data supports technique simplification, as the absence of a need for fiber use may drastically reduce the final costs. 

Sabino et al. developed an in vitro model of bioluminescent *C. albicans* 72-h biofilms inside dental curved root canals and investigated the microbial reduction using MB (90 μM; PIT: 2 min) with a diode laser device (λ = 660 nm, 100 mW, 6 min for each canal; 18 min/tooth; 36–108 J/tooth) and two light delivery systems: (1) laser handpiece tip in contact with pulp chamber access or (2) an optical diffuser fiber within the canal made of flexible polymethyl methacrylate and coated with fluorinated polymer [81]. This fiber was capable of delivering homogeneous radial light dispersion for 1.5 cm from its distal extremity and was applied with spiral movements from apical to cervical (Table 2). Disclosed outcomes showed that there was a substantial decrease in microbial load after the laser tip irradiation; nevertheless, when the irradiation was performed using the diffuser fiber, the reduction was significantly higher. The laser tip promoted a mean reduction of almost 2 log after the total treatment, while the fiber reduction achieved was over 3.5 log in the same period. As final conclusions, authors affirm that aPDT was effective with both light delivery systems, but microbial reduction was nearly 100 times more effective when irradiation was performed with a fiber [81].

Searching for aPDT studies applied to endodontics performed with or without fibers, the first aPDT report was carried out without an intracanal fiber [88], but the following ones made use of it [89,90,91] based perhaps on common sense (professionals are trained to use instruments along the whole root canal length, and a fiber is in consonance with practice standards). Studies of whether the fiber is advantageous or not only came later in 2011 [80,92]. Moreover, experiments of intracanal fiber benefits against polymicrobial biofilms are needed to fulfill this topic. 

### 7.2. Light Irradiation through Periapical Bone

In endodontics, PS activation in root canals has been settled as a critical point due to low free oxygen diffusion inside root canals, and it is well-known that aPDT is an oxygen-consuming modality. Furthermore, all previous experimental setups have in common that PSs are triggered inside root canal. Consequently, Cieplik et al. evaluated aPDT in overnight *E. faecalis* stationary-phase inactivation on the distal root canal of the first molar by external light triggered through simulated human dental hard and surrounding tissues [82]. Authors developed an experimental model with a resin composition based on data from transmission measurements of a porcine mandible with three lower human teeth (one premolar and two molars). In this ex vivo model, light activation occurred externally, and two PSs were tested at the same concentration of 10 μM: MB (Waldmann PDT 1200 L light system; λ = 570–680 nm, 4.53 J·cm^−2^, 37.8 mW·cm^−2^) and TMPyP (Waldmann Blue V; λ = 400–460 nm; 2.4 J·cm^−2^, 20 mW·cm^−2^), as described in Table 3. Both were irradiated for 120 s and buccal hemisected mandibular halves transmission measures at first molar were performed. Final outcomes revealed a successful aPDT with both PSs (mean bacterial reduction by ≥ 5 log). From transmission measurements, the shortest wavelength that penetrated dental tissue properly was 430 nm. Although the transmission at this wavelength was 0.84%, 10 μM TMPyP activation was sufficient to achieve aPDT efficacy (reduction of 6.5 log) [82].

The typical penetration or red light (λ = 630–660 nm) in living tissue used for PDT is only 1–2 mm [62], but when red light illuminates the target area, the energy of the incident light falls dramatically at increasing depths below the surface. This is because most tissue chromophores (hemoglobin, melanin, fat tissue, etc.) absorb light strongly in the visible spectrum (variables not contemplated in the experimental model of Cieplik et al.). For the deep penetration of light into living tissues (>5 mm), light should be in the near-infrared region (NIR) between 700 and 1300 nm, which is called the optical window of biological tissue [93,94]. However, Cieplik et al. showed that external light activation at a distance between 2.5 and 6 cm from outside a tooth may be possible at wavelengths ≥ 430 nm, facilitating aPDT clinical application in endodontics [82] (Table 3). Additionally, before transferring the results to clinical practice, the ex vivo outcomes must be repeated in biofilms, since bacteria present in infected root canals grow mainly as a sessile biofilm. Also, the energy absorbed per unit mass of tissue must be estimated, since there is a clear lack of a widely accepted definition of PDT light dose in the literature. 

## 8. Negative Pressure Systems

In endodontics, in order to deliver the irrigant into the root canal for the entire length and to obtain an improved fluid dynamic, negative-pressure systems have been introduced to simultaneously release and remove classical irrigants. Negative pressure creates a flow strong enough to flush out debris, preventing the irrigant from overflowing to periapical tissues [95]. The EndoVac^®^ system (Discus Dental, Culver City, CA, USA) comprises a master delivery tip with a macro and microcannula, which allows the delivery and evacuation of the solution alongside the tooth. Negative-pressure studies have shown that this technique is very effective in ensuring a greater volume of irrigant in the apical third [96,97]. Centered on this, Miranda et al. studied the ex vivo antimicrobial efficacy of the EndoVac^®^ system combined with aPDT as adjuncts to chemomechanical debridement associated with calcium hydroxide in reducing the levels of intracanal *E. faecalis* 30-day biofilms (Table 3). For aPDT procedures, MB (25 μg mL^−1^; PIT: 5 min) was irradiated by a diode laser (λ = 660 nm; 40 mW; IT: 5 min). The EndoVac^®^ system was associated with the smallest reduction (mean reduction of 0.37 × 10^2^ CFU mL^−1^), whereas the EndoVac-MB group under irradiation had the greater decrease (2.1 × 10^4^ CFU mL^−1^). The final conclusion was that EndoVac-MB-PDT was as effective as chemomechanical debridement associated with calcium hydroxide in reducing intracanal *E. faecalis* levels [83]. 

## 9. Peptides

Proteins and peptides are fundamental components of cells that carry out important biological functions. Structurally, proteins and peptides are very similar, constituted by amino acids chains that are held together by amide bonds, but peptides are smaller and with less-well-defined structure than proteins, which can adopt complex conformations. Peptides may be subdivided into oligopeptides (Ols), which have few amino acids, and polypeptides with numerous amino acids. 

### 9.1. Oligopeptides

Oligopeptides (Ols) are mainly made up of up to 50 amino acid residues, are virtually ubiquitous, and play a role in a host innate immune defense against infection due to their important role in several microorganism species’ cell-membrane disintegration [98]. Ols are usually found in prokaryotes and in eukaryotes. Natural and synthetic Ols have been investigated, but to date, none has yet emerged as a solid therapeutic agent [99]. Recently, de Freitas et al. tested aPDT enhanced by the peptide Aurein 1.2 (AU_1.2_), a helical cationic Ol from *Listeria* spp. AU_1.2_ has no stated secondary structure, but in aqueous solutions assumes an α-helical amphipathic cationic conformation that presents a rapid antimicrobial effect. De Freitas and co-workers combined 16 μM of AU_1.2_ with three distinct PSs (MB, chlorin *e*_6_, and curcumin) in an *E. faecalis* model and additional antimicrobial tests were run on extra bacteria to achieve the best antimicrobial pattern consistency *(*Table 4). The aPDT treatment with conjugates AU_1.2_–MB (156 μM; PIT: 5 min) and AU_1.2_–c*e*_6_ (84 μM; PIT: 5 min) achieved the best antimicrobial outcomes, leading to total bacterial elimination (over 10 log reduction) when samples were irradiated with red light (λ = 660 nm; 151 mW·cm^−2^) with the required total light dose of 45 and 30 J·cm^−2^, respectively. Additionally, it was possible to perceive that MB accumulated inside prokaryote cells, while chlorin *e*_6_ attached to the outside of bacterial cells. The studies revealed not only distinct mechanisms of action, but also evidenced the multiple-target nature of the photodynamic effect [100].

### 9.2. Polypeptides

As far as polypeptides are concerned, polylysines (Polys) deserve special attention. These homopolymers of lysine (an α-amino acid) with several forms of stereochemistry and link positions enable the attachment of cells and proteins to solid surfaces. In histochemical applications, both polymers of d- and l-lysine are used to coat slides to promote cell attachment. Our investigation group recently used sterile 12-well polystyrene microtiter plates with glass coverslips coated with poly-d-lysine (Sigma-Aldrich^®^, P1149, Saint Louis, MO, USA) to observe fresh cultures of 48-h mono- and dual-species biofilms of *E. faecalis* and *C. albicans* in confocal fluorescence microscopy [10]. 

In aPDT, Polo et al. used polylysine–porphycene conjugates as efficient PSs for microbial pathogen inactivation. In this study, poly-l-lysine was conjugated with two porphycenes (porphyrin electronic isomers): 2,7,12,17-tetrakis(2-methoxyethyl)-9-glutaramidoporphycene (GlamTMPn) and 2,7,12,17-tetrakis(2-methoxyethyl)-9-*p*-carboxybenzyloxyporphycene (BOHTMPn) (Figure 2). Both blue conjugates, GlamTMPn-Polys and BOHTMPn-Poly (10 μM; PIT: 30 min), were finally diluted in hydrogen chloride and tested against bacteria and fungi when irradiated with white light (λ = 650 nm; 150 mW·cm^−2^). The proposed strategy combines the main elements of the two approaches through the covalent binding of the PS to the Poly (as a cationic agent), which can alter the organization of the bacterial outer membrane (Table 4). The antimicrobial outcomes of both conjugates displayed a high efficiency in MRSA killing [101]. Later, the same group repeated the conjugates as PSs on solid media of bacteria irradiated by a 630 nm LED. Both were accumulated in significant amounts by *Prevotella intermedia*, *Peptostreptococcus micros*, and *Aggregatibacter actinomycetemcomitans*, but not by *Fusobacterium nucleatum*. However, as final conclusions, Lauro and co-workers deliberated that aPDT based on GlamTMPn–Poly exhibited a higher affinity for bacterial cells [15]. In 2017, Chen et al. used a fabric coated with a ε-polylysine (EPL) to introduce a second layer of a metallophthalocyanine, the zinc(II) complex of a mono-substituted β-carboxyphthalocyanine (CPZ). The complex obtained, named as Fabric–EPL–CPZ was tested as PS against drug-resistant bacteria with a pre-incubation period of 10 min. The final conclusions drawn were that Fabric–EPL–CPZ excited by He-Ne laser light (λ = 663 nm, 150 mW·cm^−2^; IT: 10 min) was able to reduce the survival rate of *E. coli* and of non-resistant *S. aureus* by 99% and 98%, respectively, as well as MRSA. This strategy of using antimicrobial agents is based on two confluent mechanisms: EPL is able to disrupt bacterial membranes due to the amino group’s cationic charges, while CPZ layers form ROS, and both damage bacterial cell membranes [102].

## 10. Other Approaches for Improving aPDT

In this field, several strategies have been applied to improve the efficacy of already known and tested PSs. Earlier, we reviewed and described external vehicles. In this section, we discuss other methodologies to improve PS photodynamic action efficacy (Table 5), namely, the presence of charge in the PS molecular structure and incubation time, among others. 

### 10.1. PS Structural Features

Considerable information has been written about the cation-chelating character of similar compounds in relation to the mediation of their biological action. Even with huge amounts of work on how PSs molecules are restructured (when more than two strategies are applied), no clear information is available on which strategy is the leading actor. In this field, the research line has moved away from negative to positive charges [103], regardless of PS nature, as it has been discovered that microbial infections are triggered by biofilms. Moreover, by definition, a biofilm involves single cells and microcolonies embedded in a highly hydrated and predominantly anionic EPS matrix [104]. The production of an anionic EPS-rich matrix is not an exclusive attribute of bacterial biofilm types—Gram-negative (uronic or ketal-linked pyruvates), Gram-positive (teichoic acids and proteins in the case of coagulase-negative bacterial biofilms)—it is also found in microalgae and fungi in which carbohydrates are the major components [105]. Numerous approaches have been used to modify the PS structures by introducing positive charge by several methods, since a direct relationship between the photoinactivation efficiency against *E. coli* and the number of positive charges on such PS molecules was observed [106]. It has been reported that cationic PSs are able to induce microbial photoinactivation with several charge distributions (mono-, di-, tri-, and tetra-cationic) with different outcomes [107,108,109,110].

For instance, Mesquita et al. tested the immobilization of a non-effective pyrrolidine-fused chlorin derivative Chl–TPFPP obtained from 5,10,15,20-tetrakis-(pentafluorophenyl)porphyrin on two commercial solid supports (also stated in Table 5): a 3-bromopropyl-functionalized silica (3-SiO_2_) and Merrifield resin (Mr) for the photoinactivation of Gram-negative bacteria as Chl–TPFPP/3-SiO_2_ and Chl–TPFPP/Mr, since both enable the consecutive PS recovery after aPDT. Particularly, Mr is a chloromethylated polystyrene crosslinked with 1% divinylbenzene that promotes a higher PS surface dispersion, leading to advanced positive charge. Furthermore, 1-methylimidazole and pyridine were also added to both conjugates for additional positive charges, and the immobilized conjugates for aPDT tests were under magnetic stirring. Final results showed that among all conjugates, Chl–TPFPP/Mr-Pyridine (100 μM; PIT: 10 min) was the most efficient in *E. coli* photosensitization after white-light irradiation (380–700 nm; 4.0 mW·cm^−2^) for 180 min [111]. 

Chen et al. (already described in Section 9, Polypeptides subsection) showed that amine groups covering the CPZ improved its antimicrobial efficacy [102]. In 2015, Zhang et al. successfully used a decacationic charged fullerene LC16 (see the detailed description of the study in Section 5.1 on carbon), bearing decatertiary amine chains. A high number of cationic charges incorporated on a fullerenyl monoadduct structure requires the synthesis of a hydrophilic addend containing the same quantity of cationic moieties, and based on this, authors used a well-defined water-soluble pentacationic *N,N,N,N,N,N*-hexapropyl-hexa(aminoethyl)amine arm moiety C_3_N_6_^+^ with five positive charges per arm. Subsequently, two quaternary alkylammonium units were attached to assemble a well-defined decacationic derivative. Moreover, the same author added a non-toxic potassium iodide (KI) as soluble salt (Table 5) to potentiate the antimicrobial activity by fullerenes and concluded that KI potentiated the light-mediated killing in bacteria and fungi by a factor of around 100 [59]. 

Spesia and Durantini team tested the two cationic porphyrins 5,10-bis(4-methylphenyl)-15,20-bis(4-*N*,*N*,*N*-trimethylammoniumphenyl)porphyrin (MPAP^2+^) and 5,10,15,20-tetrakis(4-*N*,*N*,*N*-trimethylammoniumphenyl)porphyrin (TMAP^4+^) as well as the anionic porphyrin 5,10,15,20-tetrakis(4-sulphonatophenyl)porphyrin (TPPS^4−^) on *E. coli* photoinactivation [112,120]. In this investigation, authors tested if the addition of divalent cations, calcium (Ca^2+^) and magnesium (Mg^2+^), improved antimicrobial efficacy. The bacteria suspensions were incubated in the presence of the PSs for 15 min in the dark before the aPDT experiments (visible light; 90 mW cm^−2^; IT: 10 min). Main assumptions were that both cations shared a similar behavior and were not toxic. Ca^2+^ and Mg^2+^ enhanced the cell uptake of MPAP^2+^ (an amphiphilic PS with two positive charges) and TPPS^4−^. In contrast, the cell uptake of TMAP^4+^ decreased (Table 5). So, the aPDT effect induced in the presence of Ca^2+^ and Mg^2+^ was only enhanced by MPAP^2+^and TPPS^4−^. Moreover, the aPDT effect seems to be dependent of the PS uptake, and it was demonstrated that the number of positive charges was an important factor in the attachment of porphyrin to microbial cells. Additionally, the divalent cations were able to enhance the antimicrobial activity of the non-positively charged TPPS^4−^ against *E. coli* [112]. This study confirms that the uptake of anionic PSs by bacterial cells may be mediated through a combination of electrostatic charge interaction and by protein transporters, while the uptake of cationic PSs is mediated by electrostatic interactions and self-promoted uptake pathways [121].

### 10.2. Incubation Period

PSs’ dark incubation period (IP), also called the pre-irradiation period, is an essential factor in promoting the contact between the microorganism cells and the PS. At present, aPDT assays must consider longer incubation times designated as dark control in order to recognize PS cytotoxicity in total absence of light [122]. The period between PS administration and shining the light named as pre-incubation time (PIT) for antimicrobial purposes should be small (few minutes) since the PS uptake by the bacteria is fast in contrast to eukaryotic cells (IP: 4–24 h) [123]. Based on this, it is mandatory to have an inert and non-toxic PS with negligible dark toxicity and strong antimicrobial action only when activated by a specific light source in the presence of molecular oxygen. Some studies affirm that a pre-incubation period improves aPDT outcomes, and available data show periods on the order of minutes, mainly 5–15 min [12,124,125]. Dark toxicity depends on PS concentration and microorganisms experimental type (i.e., biofilms or planktonic cells) [122]. Note that a greater ratio of light/dark toxicity means a more advantageous PS [126]. 

Recently, our research group quantified PSs’ dark toxicity at 0.1 mg mL^−1^ for TBO, TMPyP, Zn(II)*e*_6_Me, and RB in 48-h mono- and dual-biofilm biomasses of *E. faecalis* and *C. albicans* during an incubation period of 15 min in the total absence of light (Table 5). The single concentration was chosen based on FotoSan agent^®^ (CMS Dental, Copenhagen, Denmark). TBO and RB were purchased from Sigma Aldrich (T3260 and 330000-1G, respectively). TMPyP and Zn(II)*e_6_*Me) were prepared by our group. Outcomes of PSs’ effects in biofilm biomass in the total absence of light during an incubation time of 15 min revealed PSs’ dark toxicities with the following order TBO > TMPyP > RB > Zn(II)*e*_6_Me. The chlorophyll derivative, Zn(II)*e*_6_Me, showed the smallest activity in the dark and the best aPDT outcomes [10]. For endodontics, this parameter is a key factor for aPDT success, as it allows the PS to penetrate through dentinal tubules and to distribute inside microorganism cells, tolerating further light absorption [127]. Moreover, longer incubation periods also promote noticeable dentin discoloration when PSs are closer to the dentin–enamel interface [128]. 

### 10.3. Solubilizers

Since it was discovered that PSs aggregate easily in aqueous medium and biofilm matrix, monomeric PS form is desirable, because a high proportion of aggregated PS in water may favor the radical formation instead of singlet oxygen due to self-quenching effect in the excited state, reducing the yield of singlet-oxygen formation [129]. To improve aPDT efficacy, it is preferable to prepare PSs in their monomeric form. For this reason, generally, PSs are dissolved in deionized water, bidistilled water, buffered saline, or PBS [10,85,130]. Moreover, some authors suspended PSs in Brain Heart Infusion (BHI) broth [89] and concluded that the antimicrobial effect was lower because of the cross-linking action or the compromised half-life of singlet oxygen from proteins present in broth, and from infected root canal tissue remnants such as pulp, serum, and dentin matrix [131]. For this, several functional sets have been added to PSs to allow the bio-conjugation of moieties capable of accentuating desirable properties preventing PS aggregation [132]. 

In 2007, George and Kishen dissolved MB in four different formulations (i.e., water, 70% glycerol, 70% poly ethylene glycol (PEG), and a mixture of glycerol:ethanol:water (30:20:50) (MIX)) with detailed description of photobiological characteristics—photophysical and photochemical. MB affinity for *E. faecalis* and *A. actinomycetemcomitans* was higher for MB–water; but the conjugate MB–MIX showed the best photooxidation potential, hypothetically due to the presence of ethanol (a less-polar solvent) [113]. Authors concluded that MB photochemical properties are highly influenced by the formulation (Table 5). One year later, the same co-workers tested with the same PS how the addition of hydrogen peroxide H_2_O_2_ (an oxidant) and perfluorodecahydronaphthalene (oxygen carrier) affected the aPDT against 10-week-old *E. faecalis* biofilms, and concluded that both formulations disrupted the *E. faecalis* biofilm matrix and promoted bacterial cell inactivation [114]. 

Maisch et al. studied a commercial preparation of hematoporphyrin oligomers with no monomer or dimer molecules Photosan (SeeLab F&E GmbH, Wesselburenerkoog, Germany) dissolved in bidistilled water against *S. mutans* and *E. faecalis*. Photosan is a hematoporphyrin. Fotosan^®^ agent (CMS Dental, Copenhagen, Denmark) is a toluidine blue-O dye. In the presence of *A. actinomycetemcomitans*, 10% EDTA was added prior to illumination to observe if Photosan action was improved. Photosan was effective against *S. mutans* and *E. faecalis* with > 3 log_10_ reduction, but was not efficient against Gram-negative bacteria. However, in the presence of EDTA, Photosan had a strong effect on *A. actinomycetemcomitans* since the metal chelator is able to cause loss of microorganism cell viability and biofilm structure disruption, and can increase PS permeability. Maisch and collaborators showed that EDTA stimulated porphyrin translocation through the outer membrane of Gram-negative bacteria, promoting the aPDT effect [115]. For the present authors, it is important to distinguish the field of PS solubilizer solutions from studies in which several endodontic classical irrigants are added to improve the aPDT, such as sodium hypochlorite [133], chlorhexidine [134], and other solutions [135]. 

Recently, Hamblin and Abrahamse focused on the addition of several inorganic water-soluble salts to improve PDT [123]. Sodium azide (NaN_3_) is widely used to inhibit aPDT because it is a well-known physical quencher of singlet oxygen (^1^O_2_), and the quenching rate constant depends on mixture configuration and solvent type [136], especially when PDT is used to kill bacteria in suspension. Surprisingly, in 2012, Huang and team-workers showed that NaN_3_ addition to MB potentiated the aPDT effectiveness not only towards Gram-positive, but also Gram-negative bacteria when irradiated with a red light source (660 nm; 100 mW·cm^−2^; 8 J·cm^−2^), as described in Table 5. In the same study, polyethylenimine (PEI) and chlorin *e*_6_ (c*e*_6_) were also used, and a divergent effect was observed (for PEI–c*e*_6_, NaN_3_ decreased the antimicrobial efficacy). In contrast, with azide, aPDT mediated by MB did not require oxygen since the hydroxyl radicals formed from excited MB (an electron donor) and oxygen allowed electron transfer from azide radical and hydroxide anions. Moreover, the spin-trapping experiments demonstrated that the azidyl radical (N_3_^•^) could also be generated by photoexcited MB with no oxygen [117], and these findings lead to the possibility that in the near future aPDT might be useful for the treatment of anaerobic infection. 

In 2013, thiocyanate (SCN^-^) salt was added to MB (10 μM) to test if its efficacy was improved against *S. aureus* and *E. coli*. In solution, MB–SCN^-^ mutually produced sulfite and cyanide anions, and the addition of each salt separately enhanced aPDT bacteria killing (Table 5). The final outcomes revealed that SCN^−^ enhanced aPDT killing of both bacterial strains in a concentration- and light-dependent manner, since SCN^−^ alone was nontoxic at all concentrations for both species. Moreover, authors revealed that oxygen is indispensable for these experiments, since O_2_-free irradiated MB samples were incapable of killing bacteria even in the presence of SCN^−^ [118]. 

Huang et al. recognized that the aPDT efficacy of non-cationic porphyrins such as Photofrin against Gram-negative bacteria and fungi was tremendously low. Centered on this, Huang and co-workers showed that the addition to Photofrin (200 nM) of the non-toxic inorganic salt potassium iodide (KI) at a concentration of 100 mM improved aPDT with remarkable bacterial eradication outcomes in five different species with >6 log killing (with minor differences in susceptibility with the order being *A. baumannii* > *E. coli* > *K. pneumoniae* > *P. mirabilis* ≈ *P. aeruginosa*). Gram-positive MRSA and *C. albicans* aPDT eradication was also potentiated by KI (Table 5). Moreover, the author observed that photochemical mechanisms were distinctive according to the species. Particularly, in Gram-negative bacteria, the microorganism suppression results from the generation of microbicidal molecular iodine (I_2_/I_3_) and its production was dependent on singlet oxygen production by PS light activation. In MSRA, the potentiation by KI might be facilitated by short-lived iodine reactive species and in *C. albicans* killing occurs via singlet oxygen (^1^O_2_) [119]. Similar effects were also observed with cationic porphyrins [137].

## 11. Sonodynamic Therapy

Sonodynamic therapy (SDT) is an attractive and additional technique for sensitizer activation that does not use an external light source. SDT relies on the generation of ROS through sonodynamic excitation induced by low-intensity ultrasound of several compounds called sonosensitizers (SSs) in the presence of molecular oxygen [138,139]. The precise mechanism of SDT remains unknown, although it seems feasible that numerous sonosensitization mechanisms operate according to diverse SSs classes [140,141].

Possible theories of SDT mechanisms include ROS generation (similar to aPDT), ultrasonic acoustic cavitation, and thermal destruction [142]. Presently, the most acceptable mechanism relies on ROS production which causes apoptosis [142]. An alternative theory advocates that the elevated temperatures lead to thermal destruction and might also result in the generation of free radicals directly from the sensitizers [140], with ROS being successively formed in an acoustic cavitation phenomenon—a similar approach to a PDT type I reaction [143]. When compared to aPDT, SDT’s main advantage is that ultrasound propagates deeper than light, and therefore may be used to treat diseases mediated by complex and dense biofilms. Moreover, SDT may facilitate the generation of transient pores in the biofilm matrix, enabling a greater diffusion of PSs, SSs, and light [143], or as a complement to aPDT [142] since the main advantage of SDT over aPDT is that ultrasound can be tightly focused with penetration in soft tissue up to several tens of centimeters. 

In 1989, Yumita et al. showed that hematoporphyrins and xanthene dyes (specially RB), commonly used as PSs can enhance the antitumoral effect of ultrasound, suggesting SDT as a new modality for treating tumors [144]. Since then, RB has been studied as a sensitizer in both approaches in several applications [145]. One issue that remains is the design of an appropriate ultrasonic device to activate the SSs at the precise frequency to optimize the SS effect. To date, the majority of studies have used laboratory and clinical instruments adapted to the main study goal.

Briefly, SDT is based on ultrasound energy, and in terms of the 3 nomenclature in this paper, it is divided into ultrasound activation (<1 MHz) and ultrasonic sonication (≥1 MHz). In ultrasonic activation, lower frequency (20–50 kHz) has been applied to sensitizers in aqueous solutions for endodontic purposes. In opposition, ultrasonic sonication links to higher frequencies applied by instruments that promote total sample vibration. 

### 11.1. Ultrasonic Activation

In 2013, Nakonechny et al. demonstrated for the first time that SDT was effective in Gram-positive and Gram-negative bacteria eradication with RB activated at 28 kHz at an intensity of 0.84 W·cm^−2^ in the total absence of light (Table 6). MB was also tested but was unsuccessful, since sonoluminescent light has a broadband spectrum from 200 to 700 nm, but the maximum emission intensity of sonoluminescence in water is 250–600 nm [146]. This emission range correlates with the RB absorbance spectrum, but has no overlap with the MB spectrum, clarifying the high rate of RB activation by SDT [147]. 

Ghinzelli et al. in an in vitro study evaluated passive ultrasonic irrigation (PUI) using a device that is already available in the Brazilian market, Nac Plus™ ultrasound (38 kHz–Adiel, Ind. e Com., Ribeirão Preto, Brazil), to activate MB over a root canal system infected with *E. faecalis* for 30 days [150]. PUI was first portrayed by Weller et al. [116], but the term “passive” does not adequately define the process, as it is in fact active. It is related to the noncutting action [151]. Samples were distributed into five groups (*n* = 10): G_1_ control; G_2_ MB as PS; G_3_ MB activated by Nac Plus™ ultrasound for 60 s; G_4_ MB activated by a red 100-mW GaAlPln diode laser (GaAlPln, 660–690 nm) for 90 s; and finally, G_5_ with aPDT + SDT (Table 6). As main conclusions, the use of MB alone, with, or with no ultrasonic activation has resulted in the worst levels of decontamination when compared to groups where MB was irradiated. Moreover, there was no statistically significant difference between groups G_2_ and G_3_. G_5_ (aPDT + SDT) revealed the best outcomes, reducing *E. faecalis* content from the root canal space, probably leading to a strong physical effect against bacterial biofilms, since it was outside of the sonoluminescent light broadband spectrum [151]. Later in 2015, Tennert et al. tested another endodontic ultrasonic device, under the commercial name of VDW Ultra Device (VDW, Munich, Germany) with an ultrasound range of 28–36 kHz to activate TBO with chelating agents to disintegrate *E. faecalis* 72-h biofilms and remove the smear layer from artificially infected root canals [151]. These authors tested several approaches within 10 experimental groups (single and multiple variables)—a strategy that has been currently replicated to improve aPDT efficacy with mixed protocols and numerous antimicrobial techniques [148,151].

In summary, from all groups, TBO with a lower pre-incubation period (60 s), combined with endodontic solutions (20% of EDTA or 20% citric acid solution) or activated by PUI did not improve the final outcomes as its sonodynamic activation was not observed (Table 6). The classical endodontic irrigant, 3% NaOCl, achieved the highest antimicrobial effect [148]. 

### 11.2. Ultrasound Sonication

Under ultrasound sonication, Wang et al. preformed an MRSA inactivation by curcumin (Table 6). The author and collaborators used 40 μM of curcumin incubated in the dark for 50 min. Subsequently, the 24-well plate was fixed on a platform in an acrylic water tank containing degassed water and exposed to ultrasound (1 MHz; 1.56 W·cm^−2^) in continuous waves for 5 min. As final results, curcumin activated by sonication promoted MRSA eradication in a curcumin-dose-dependent manner with 5 log reduction in CFU [149].

The ability of aPDT, SDT, and the combination aPDT + SDT were similarly evaluated in eukaryotic organisms. Alves et al. used RB and chlorin *e*_6_ derivative Photodithazine^®^ (PDZ), an *N*-methylglucosamine derivative of chlorin *e*_6_. Both PSs were diluted in physiological saline and the eradication efficacy was evaluated after a pre-incubation period of 30 min in *C. albicans* planktonic suspensions and biofilms. For this, RB was irradiated for 5 min with a white light and PDZ by a red light. Sonication was provided for 5 min by a Sonidel SP100 sonoporator (Sonidel Ltd, Dublin, Ireland), at a frequency of 1 MHz and a pulse repetition frequency of 100 Hz. Combined treatments were applied simultaneously (aPDT + SDT). In the experiments, while aPDT and SDT eradicated planktonic cells separately, both had low impact on biofilms. An important aspect to mention is that PDZ was excited by a light with proper wavelength (660 nm) and RB (λ = 549 nm) was not, and this might influence the final aPDT efficacy. Still, aPDT + SDT showed a significant cell viability reduction, and reduced total biofilm biomass (thinner biofilms comprised mainly of dead cells) [143]. It was hypothesized that both approaches were able to disrupt the biofilms, enabling PS penetration deep into the biofilms since ultrasound increases the uptake of molecules through microorganisms’ membranes’ transient pores (via sonoporation). Another possibility is that the physical agitation from the ultrasound provokes microorganisms’ faster circulation in the wells, enhancing the light exposure [143].

## 12. Conclusions

Antimicrobial PDT (aPDT) is a current strategy to effectively combat a broad spectrum of biofilm-mediated diseases. This approach presents particular interest in endodontics due to the exceptional anatomical variations in human tooth morphology, which preclude the effective biofilm mechanical disruption inside the root canal system and dentinal tubules. Moreover, to overtake the limitations of lower molecular oxygen availability inside the root canal system, several approaches (some of them at the experimental level) were reviewed here to provide guidance for aPDT efficiency improvement. It is essential to understand these chemical principles and aPDT mechanisms because in microbial cells, outer membrane damage plays an imperative role. This is in contrast to mammalian cells, where the main targets of aPDT are lysosomes, mitochondria, and plasma membranes. 

From all appraised approaches that could be conjugated with several PSs in order to optimize their properties and effects, the most quoted and tested to date are (i) drug delivery systems (especially the nanoparticles field) and (ii) PS solubilizers. These two methodologies might be theoretically implemented in endodontics with promising results, since both of them have already been tested in in vitro endodontic biofilms. 

Currently, it is important to insist that the main aPDT focus remains on finding the ideal non-toxic PS combined with an accurate light wavelength that excites the PS to its best performance. The ongoing PS development is being carried out with several techniques in order to potentiate its antimicrobial breadth of action to a maximum, increasing its efficacy with no microorganism-induced resistance mechanism. 

While basic research suggests that there is still room for improvement on multiple fronts, preclinical evidence is scarce and limited to a small cluster of photosensitizers that weakly bind to the microorganisms’ membrane, such as TBO and MB. Remarkably, some technologies have been combined with the predilection of PS recuperation from the host environment after its chemical consumption, with a focus on re-application in a sustainable and ecological approach with no toxic effects to the host. This might be worthwhile for endodontics, since endodontic infections are related to infected root canal systems as well as periapical tissues, and it is mandatory to have a non-biofilm area to ensure periapical tissue healing. 

The application of additional photoactive materials in aPDT opens new opportunities for improvement as well as the development of synergistic approaches to root canal disinfection, in order to overcome its current underuse in clinical settings.

## Figures and Tables

**Table 1:** Bacteriophage and drug delivery systems characteristics used with focus on aPDT improvement.

System		Approach	PS/λ_(nm)_	Microorganism(s)
**Bacteriophage [19]**		Supramacromolecule of DNA and Coated Proteins	**PPA** 658	*Candida albicans*
**Drug Delivery Systems**	**Cellulose** [23,24,25]	Cellulose acetate dissolved in acetone	**TBO** **RB** 610/545	MRSA, *Escherichia coli,* *Clostridium difficile,* *Bacteriophage, and* *C. albicans*
		Cellulosic fabric of β(1,4)-d-glucopyranose chains	**TMAP ^4+^** **ZnTMAP ^4+^**	*Staphylococcus aureus, E. coli,* and *Pseudomonas aeruginosa*
	**Chitosan** [26]	Poly-β(1,4)-d-glucopyranosamine	**RB** **MB** 540	*Enterococcus faecalis* *P. aeruginosa*
	**Alginate Foam/** **Cyclodextrins** [27,28]	Foam formulations constituted by the gel-forming polymer sodium alginate, the gelling agent calcium carbonate, the plasticizers sorbitol and glycerol, the foaming agent hydroxypropylMethylcellulose, and as PS solubilizer agents β- and γ-cyclodextrins and polyethylene glycol 400	**Curcumin**	Infected wounds
	**Electrolyzed Water (EW)** [29]	Water and salt (sodium chloride). Acid–EW and Alkaline–EW	**RB Erythrosine Phloxine**	*Streptococcus mutans*
	**Hydrogel** [30]	Cross-linked poly (vinyl alcohol) (PVA)–borate complexes	**MB** **TMP** 635	MRSA
	**Lipid Delivery System** [31,32,33]	**Invasome**	**mTHPC**652	*E. faecalis*
		**Liposomes**	**TDPyP**	MRSA
		**Micelles**	**Hp** 635	MRSA *Staphylococcus epidermis* *Streptococcus pyogenes*
	**Oil Based Emulsions** [34,35]	**Microemulsions**	**TBO** 600	*P. aeruginosa*
		**Nanoemulsions**	**ClAlPc** 660	Methicillin-susceptible *S. aureus* and MRSA

**LEGEND—**PPA: pheophorbide *a*; TBO: toluidine blue-O; RB: rose bengal; TMAP^4+^: 5,10,15,20-tetrakis(4-*N,N,N*-trimethylammoniumphenyl)porphyrin; ZnTMAP^4+^: zinc(II) complex of TMAP^4+^; MB: methylene blue; TMPyP: 5,10,15,20-tetrakis(1-methylpyridinium-4-yl)porphyrin tetra-tosylate; MRSA: methicillin-resistant strain of *Staphylococcus aureus*; mTHPC: 5,10,15,20-Tetrakis(*m*-hydroxyphenyl)chlorin; TDPyP: 5-(1-dodecanoylpyridinium-4yl)-10,15,20-triphenylporphyrin; Hp: hematoporphyrin dihydrochloride; ClAlPc: chloro-aluminumphthalocyanine.

**Table 2:** Metal–organic frameworks (MOFs), the nanoparticle systems most used to improve aPDT efficacy to date, and their features.

System		Approach		PS/λ_(nm)_	Microorganism(s)
**Metal–Organic Frameworks (MOFs) [54]**		Metal ions coordinated to organic ligands with one-, two-, or three-dimensional structure		ICG 810	*E. faecalis*
**Nanoparticles** **(NPs)**	**Carbon** [55,56,57,58,59]	Carbon Nanotubes	SWCNTs	H_2_TriMAPP 419	*S. aureus*
			MWCNTs	PPIX	
		Nano-graphene oxide (NGO)		ICG 810	*E. faecalis*
		Fullerenes (C_60_, C_70_, and C_84_) in a closed sphere of carbon molecules		BF4-6	*S. aureus*, *E. coli*, *C. albicans*, *P. aeruginosa*
				LC16	*Acinetobacter baumannii,* MRSA, *C. albicans*
	**Chitosan** [26]	Poly(d-glucosamine)		MB RB 540	*E. faecalis*
	**Gold** [60]	Colloidal gold particles complexed with poly lactic-*co*-glycolic acid (PLGA)		MB 665	
	**Platinum** [61]	Platinum hexagonal nanoparticles		ClGaTCPP	*S. aureus*
	**Silica** [62]	Pure SiO_2_ nanoparticles synthesized by hydrolysis of tetraethyl orthosilicate in reverse microemulsion		RB 525	MRSA, *S. epidermis*
	**Silver** [63]	Silver nitrate was dissolved in *n*-methylpyrrolidone and mixed with solution of PMMA in dichloroethane		TPP 405/470	*P. aeruginosa,* *S. aureus*
	**Superparamagnetic Iron Oxide (SPIONs)** [64]	Hematite (α-Fe_2_O_3_), maghemite (γ-Fe_2_O_3_), and magnetite (Fe_3_O_4_)			*S. aureus, S. mutans,* *E. coli*

**LEGEND—**SWCNTs: single-walled carbon nanotubes; MWCNTs: multi-walled carbon nanotubes; TMAPP: 5,10,15-triphenyl-20-(4-aminophenyl)porphyrin; PPIX: protoporphyrin IX; ICG: indocyanine green; BF1–3: functionalized C_60_ with one, two, or three polar diserinol groups; and BF4–6: functionalized C_60_ with one, two, or three quaternary pyrrolidinium groups; LC16: fullerene-functionalized C_60_; ClGGaTCPP: 5,10,15,20-tetrakis-(4-carboxyphenyl)porphyrinate gallium(III) chloride; TPP: 5,10,15,20-tetraphenylporphyrin.

**Table 3:** Efflux pump inhibitors (EPIs), light source, and negative pressure system features presumed to improve aPDT efficacy.

System		Approach		PS/λ_(nm)_	Microorganism(s)
**Efflux Pump Inhibitors (EPIs) [78,79]**		**NorA**	Deficient mutants of Gram +	**TBO** **MB** **DMMB** ****pL**–c*e*_6_** 660	*S. aureus*
		**TolC**	Deficient mutants of Gram −		*E. coli*
		**MexAB**			*P. aeruginosa*
		**Verapamil**		**MB** 660	*E. faecalis*
**Light Delivery Systems**	**Optical fiber** [80,81]	Optical fiber inside the root canal at the established working length (WL) with spiral movements for apical to cervical		**MB** 660	
		Optical diffuser fiber within the canal			*C. albicans*
	**Through Periapical Bone** [82]	Experimental model with human premolars and molars in an acrylic resin bloc simulating the optical properties of a porcine jaw		**TMPyP^b^** **MB** 430	*E. faecalis*
**Negative Pressure System** [83]		EndoVac^®^ system (Discus Dental, Culver City, CA, USA)		**MB** 660	*E. faecalis*

**LEGEND—**TBO: toluidine blue-O; DMMB: 1,9-dimethylmethylene blue; pL–c*e*_6_: poly-l-lysine–chlorin *e*_6_ conjugate; TMPyP^b^: 5,10,15,20-tetrakis(1-methylpyridinium-4-yl)porphyrin tetra-(*p*-toluenesulfonate).

**Table 4:** Peptide approaches used to improve aPDT efficacy and their features.

Systems		Approach	PS/λ_(nm)_	Microorganisms
**Peptides**	**Oligopeptides** [100]	Aurein 1.2 (AU_1.2_) peptide with 13 amino acid residues	**MB** **Chlorin *e*_6_** **Curcumin** 660	*E. faecalis, S. aureus,* *A. baumannii, E. coli,* *Enterococcus faecium,* VRE
	**Polypeptides** [101,102]	Poly-l-lysine hydrochloride added to porphycenes	**BOHTMPn** **GlamTMPn** 650	*E. coli,* MRSA, *C. albicans*
		ε-Polylysine acquired from a commercial department	**CPZ** 630	*E. coli*, *S. aureus* (two strains of non-resistant and one resistant to methicillin)

**LEGEND—**BOHTMPn: 2,7,12,17-tetrakis(2-methoxyethyl)-9-*p*-carboxybenzyloxyporphycene; GlamTMPn: 2,7,12,17-tetrakis(2-methoxyethyl)-9-glutaramidoporphycene; CPZ: mono-substituted β-carboxyphthalocyanine zinc(II); VRE: Vancomycin-Resistant Enterococci

**Table 5:** Other approaches used to improve aPDT efficacy.

Approach		Materials or Methodologies		PS/λ_(nm)_	Microorganism(s)
**OTHER APPROACHES FOR IMPROVING aPDT**	**PS Structural Features** [59,102,111,112]	**3-Bromopropyl functionalized silica** and **Merrifield resin** positively charged with **1-methylimidazole** and **pyridine**		**Chl-TPFPP**	*E. coli*
		**Amine groups** and **chains** as coupling chemistry		**LC16**	*Acinetobacter baumannii,* MRSA, *C. albicans*
		**Divalent cations** such as Ca^2+^ and Mg^2+^ from CaCl_2_ and MgCl_2_		**TMAP^4+^** **MPAP^2+^** **TPPS^4−^**	*E. coli*
	**Incubation Period** [10]	**Incubation periods of 5–15 min** accordingly to several studies.		**TBO** **RB** **TMPyP** **Zn(II)*e*_6_Me** 557/627	*C. albicans,* *E. faecalis,* dual-species biofilms (Ca:Ef)
	**Solubilizers** [86,113,114,115,116]	**20% Citric Acid**		**TBO**	*E. faecalis*
		**70% Glycerol, 70% PEG,** **MIX** (glycerol:ethanol:water 30:20:50), and **H_2_O**		**MB**	*E. faecalis,* *Aggregatibacter actinomycetemcomitans*
		**BHI Broth**		**MB**	*Porphyromonas gingivalis, Peptostreptococcus micros,* *Prevotella intermedia; E. faecalis, Fusobacterium nucleatum, Porphyromonas endodontalis*
		**EDTA**	10%	**Photosan**	*S. mutans, E. faecalis,* *A. actinomycetemcomitans*
			20%	**TBO**	*E. faecalis*
		**H_2_O_2_** and **Perfluorodecahydronaphthalene**		**MB**	
		**Inorganic Salts** [81,117,118,119]	Potassium iodide (**KI**)	**LC16**	*A. baumannii,* MRSA, *C. albicans*
				**Photofrin**	*E. coli, P. aeruginosa, Klebsiella pneumoniae, Proteus mirabilis, A. baumannii,* MRSA, *C. albicans*
			Potassium thiocyanate (**KSCN**)	**MB**	*S. aureus,* *E. coli*
			Sodium azide (**NaN_3_**)	**MBc*e*_6_** 660	

**LEGEND—**Chl-TPFPP: pyrrolidine-fused chlorin derivative chlorin-2; MPAP^2+^: 5,10-di(4-methylphenyl)-15,20-di(4-*N,N,N*-tri-methylammoniumphenyl) porphyrin; TPPS^4−^: 5,10,15,20-tetrakis(4-sulphonatophenyl) porphyrin; Zn(II)*e*_6_Me: Zn(II)chlorin *e*_6_ methyl ester; PEI-c*e*_6_: polyethylenimine and chlorin *e*_6_.

**Table 6:** Sonodynamic therapy factors and individual features used to improve aPDT efficacy.

Approach		Conditions	PS/λ_(nm)_	Microorganism(s)
**Sonodynamic Therapy (SDT)**	**Ultrasonic Activation** [116,147,148]	28 kHz	**RB** **MB**	*S. aureus,* *E. coli*
		28–36 kHz VDW Ultra-Device	**TBO**	*E. faecalis*
		Passive ultrasonic irrigation (PUI)	**MB** 660–690	
	**Ultrasound Sonication** [143,149]	1 MHz	**Curcumin**	MRSA
			**PDZ** **RB** 660	*C. albicans*

**LEGEND—**Photodithazine (**PDZ**).
